# Resonant X-ray emission with a standing wave excitation

**DOI:** 10.1038/srep22648

**Published:** 2016-03-03

**Authors:** Kari O. Ruotsalainen, Ari-Pekka Honkanen, Stephen P. Collins, Giulio Monaco, Marco Moretti Sala, Michael Krisch, Keijo Hämäläinen, Mikko Hakala, Simo Huotari

**Affiliations:** 1 Department of Physics, University of Helsinki, P. O. Box 64, FI-00014, Finland; 2 Diamond Light Source, Didcot, Oxfordshire, OX11 0DE, UK; 3 Physics Department, University of Trento, Via Sommarive 14, 38123 Povo (TN), Italy; 4 ESRF - The European Synchrotron, CS40220, 38043 Grenoble Cedex 9, France

## Abstract

The Borrmann effect is the anomalous transmission of x-rays in perfect crystals under diffraction conditions. It arises from the interference of the incident and diffracted waves, which creates a standing wave with nodes at strongly absorbing atoms. Dipolar absorption of x-rays is thus diminished, which makes the crystal nearly transparent for certain x-ray wave vectors. Indeed, a relative enhancement of electric quadrupole absorption via the Borrmann effect has been demonstrated recently. Here we show that the Borrmann effect has a significantly larger impact on resonant x-ray emission than is observable in x-ray absorption. Emission from a dipole forbidden intermediate state may even dominate the corresponding x-ray spectra. Our work extends the domain of x-ray standing wave methods to resonant x-ray emission spectroscopy and provides means for novel spectroscopic experiments in d- and f-electron systems.

The pursuit of understanding the microscopic origins of the properties of bulk matter often relies on spectroscopy and scattering of x-ray photons. X-ray absorption and emission spectroscopies probe the unoccupied and occupied electronic states, respectively^1^,^2^. Magnetic circular and linear as well as natural circular dichroism in x-ray absorption spectra give access to ground state spin and angular momentum expectation values, the spin-orbit coupling constant and structural information^3^,^4^,^5^,^6^. Resonant x-ray emission and resonant inelastic x-ray scattering spectroscopies yield element, spin and orbital selective electronic structure information and probe valence electron excitations^7^. A much utilized advantage of resonant x-ray emission spectroscopy over traditional x-ray absorption spectroscopy is the ability to resolve lifetime broadening limited features in the x-ray absorption spectrum^7^,^8^. It can be stated that the introduction of resonant x-ray spectroscopies turned over the notion that the core hole lifetime is a fundamental limit to the energy resolution obtainable in x-ray absorption spectra.

On the other hand, x-ray standing wave techniques provide an attractive approach to achieve site sensitive spectroscopic information^9^. For bulk solids, applications of standing wave methods have included e.g. locating impurity sites and solving the phase problem of x-ray diffraction^9^,^10^,^11^,^12^. There also are several varieties of standing wave spectroscopies applicable to thin films, multilayers and surfaces, most impressive demonstrations of which include the determination of adsorption sites on surfaces^9^,^13^,^14^,^15^. X-ray spectroscopies and standing wave fields can thus be joint in very fruitful combinations that yield novel information on materials relevant for physics and chemistry.

Under the diffraction condition, the incident and diffracted waves in a crystal form a coherent superposition, with a standing wave along the scattering vector^16^. The relative amplitudes and phases of the waves depend on the deviation from the Bragg angle. In the two-beam case with monochromatic linearly polarised plane waves, the Maxwell equations have two solutions per polarization state with respect to the scattering plane (parallel *π*, perpendicular *σ*). One of the solutions (so-called *α*-branch) has nodes on atomic planes, and the other (*β*-branch) corresponds to antinodes at those planes. The absorption owing to the electric dipole (E1) term depends on the field amplitude at the atomic sites and thus the attenuation of the *α*-branch is diminished while the *β*-branch, in turn, is absorbed rapidly^16^,^17^. In the Laue (transmission) geometry, exciting the *α*-branch gives rise to a well known manifestation of dynamical diffraction: the anomalous transmission of x rays known as the Borrmann effect^16^.

There are fascinating ways to use the Borrmann effect, e.g., in x-ray absorption spectroscopy^17^. For instance, Pettifer *et al.* demonstrated using the Borrmann effect a very large relative enhancements of electric quadrupole (E2) resonances at the Gd L edges in Gd_3_Ga_5_O_12_, where the normally weak quadrupole-allowed pre-edges almost reached the intensity of the dipolar main edges at the temperature of 10 K^17^. Identification of the transition multipolarity is of utmost importance in the interpretation of magnetic x-ray dichroic spectroscopies and their study provided unambiguous proof of the quadrupolar barely visible pre-edge at the Gd L_3_ edge. This solved a long-standing issue of the nature of the pre-edge resonance, which had been a much discussed topic in the context of x-ray circular magnetic dichroism of rare earth compounds^18^,^19^,^20^,^21^,^22^.

One way to gain insight to their dipolar and quadrupolar contributions to x-ray spectra would be to utilise their different angular dependencies. However, such an analysis may require support from parameter-dependent crystal or ligand field calculations. Moreover, utilising the angular dependence of the multipoles is complicated in certain systems where they have similar angular behaviour^3^,^23^. The Gd^3+^ ion in GGG is one well known case of where alternative methods were needed to understand the absorption edge structure. To this end, Krisch *et al.* applied resonant x-ray emission spectroscopy to GGG and provided strong evidence for the quadrupolar nature on the pre-edge feature at the Gd L_3_ edge^23^. They measured the intensity of the Gd L*α*_1_ emission line while tuning the incident x-ray energy across the Gd L_3_ edge, revealing the quadrupole-excited state via its fingerprint in the emission spectrum.

In this work, we combine for the first time resonant x-ray emission spectroscopy and the Borrmann effect, using the Gd L_3_ resonance in GGG. We exploit the effective transparency of the crystal to the anomalously transmitted x-rays in a novel experiment that allows us to enhance the quadrupole-allowed states in the resonant x-ray emission spectra. We demonstrate a tenfold increase in the relative weight of x-ray emission from a dipole-forbidden intermediate state at the Gd L_3_ edge when excited by anomalously transmitted light. Our results thus show that high resolution x-ray spectroscopy can be successfully augmented with standing wave techniques with promising applications.

## Results

**Figure 1:**
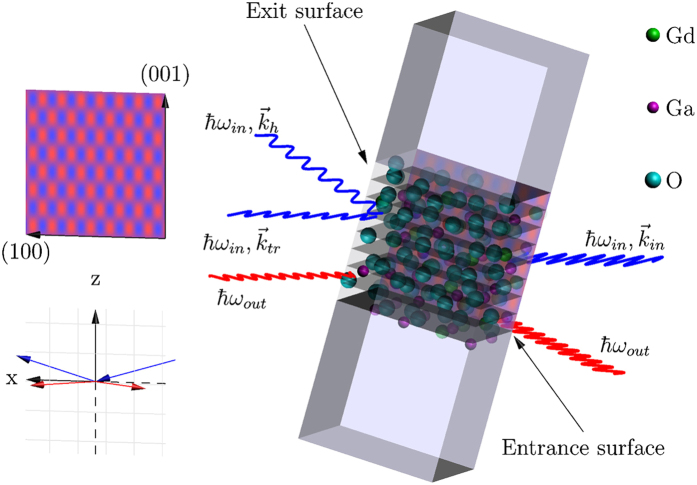
Illustration of the experiment. Top left: The wavefield pattern with respect to the (100) and (001) lattice directions in the conventional cubic unit cell of GGG. The standing wave is formed along the (001) direction. Bottom left: The diffraction and fluorescence observation geometries. The xz-plane is the vertical scattering plane, in which the anomalously transmitted beams are observed. The spectrometer observes fluorescence in the xy plane, and is rotated about the z axis for observing the emitted radiation from the entrance and exit surfaces of the sample. Right: The crystal structure of GGG and the (008) lattice planes are visualized in the center. Blue wavy lines represent the incident, transmitted and diffracted photons with E_in_ = *ħω*_in_ and wave vectors 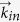, 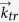, and 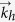. The incident beam impinges on the entrance surface (right-hand side of crystal). In anomalous transmission the energy carried by the incident beam flows along the diffracting planes and two beams emerge from the exit surface (left-hand side of the crystal). The redshifted fluorescence photons (E_out_ = *ħω*_out_) are represented by the red lines.

**Figure 2:**
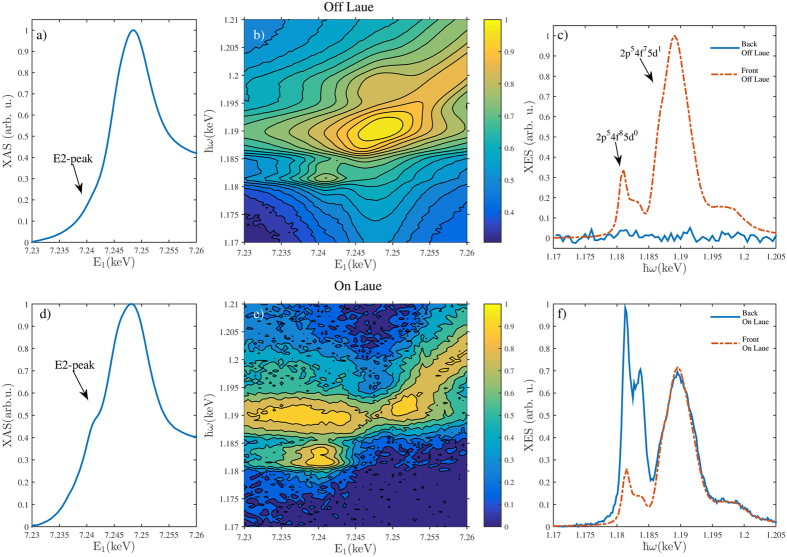
(**a**) Entrance surface off-Laue total fluorescence yield x-ray absorption spectrum of the Gd L_3_ edge, (**b**) the associated entrance surface resonant x-ray emission map on a logarithmic scale to highlight the weak emission lines, (**c**) the corresponding resonant x-ray emission spectrum excited at the pre-edge (*E*_in_ = 7.2405 keV). The solid and dashed lines denote spectra recorded from the entrance and exit surfaces of the crystal, respectively. (**d**) The transmission x-ray absorption spectrum under the diffraction condition. (**e**) Exit-surface resonant x-ray emission map on a logarithmic scale. Note the large difference in comparison with Fig. 2b. The low intensity regions of the map have been smoothed for visual clarity. (**f**) The resonant x-ray emission spectrum under the diffraction condition for *E*_in_ = 7.2405 keV. X-ray emission from the quadrupole-allowed 4f^ 8^5d^0^ intermediate state gains more weight than the dipole-allowed 4f^ 7^5d^1^ one.

We performed a resonant x-ray emission experiment on a GGG single crystal to study the emission spectrum under the Borrmann effect. We tuned the incident x-ray energy *E*_in_ across the Gd L_3_ edge and for each energy measured the resonant Gd L*α*_1_ emission (spectrometer recording intensity at a scattered photon energy *E*_out_). The energy transfer in the process is denoted as *ħω* = *E*_in_ − *E*_out_. The spectra were recorded from the x-ray illuminated entrance surface and from the exit surface of the crystal as depicted in Fig. 1. We repeated the experiment both off and on the the Laue diffraction condition, and denote the obtained spectra as *off/on-Laue spectra* which are presented in Fig. 2a–c and in Fig. 2d–f, respectively. The statistical uncertainties at the emission peak maxima in the RXES maps in Fig. 2b,e were of the order of 1% and 10% for the entrance and exit surface spectra, respectively. In Fig. 2a we present the off-Laue total fluorescence yield spectrum measured from the entrance surface. The main edge displays a nearly indistinguishable low-energy shoulder at E_1_ = 7.2405 keV, which is the quadrupole-allowed pre-edge discussed in the introduction. The main edge reaches its maximum intensity at 7.250 keV beyond which there is very little fine structure, and this region is denoted as the post edge. The corresponding entrance surface resonant x-ray emission map is shown in Fig. 2b, and displays distinct behaviour in the pre-, main- and post edge regions. One can clearly observe two multiplet families associated with the 2p^5^4f^ 8^5d^0^ (pre-edge) and 2p^5^4f^ 7^5d^1^ (main edge) intermediate states, which are the most relevant for *Lα*_1_ emission. The different Coulomb interaction energies in the final states result in an approximate 8 eV separation between these multiplets. From the entrance surface spectrum in Fig. 2c we observe that the 3d^9^4f^ 8^5d^0^ final state results in two emission peaks separated by approximately 2 eV. Their intensities are maximized when the incident-x-ray energy matches the pre-edge energy of 7.2405 keV [Fig. 2b]. On the other hand, the 3d^9^4f^ 7^5d^1^ peak at the high-energy side of the emission spectrum gains its maximum when the energy of the incident x-rays corresponds to the main edge, i.e. 7.250 keV. Past the main edge, the emission spectrum is dominated by final states where the excited electron is promoted into the continuum, resulting in a feature that disperses linearly with the incident energy in resonant x-ray emission, leading into off-resonant emission behaviour, i.e., x-ray fluorescence.

The off-Laue RXES spectrum presented in Fig. 2c taken from the exit surface obviously displays only background noise, because the x-rays are strongly attenuated within the crystal and its back side appears as dark in the x-ray wavelengths. The off-Laue emission spectrum from the entrance surface, on the other hand, displays two major structures. A weak double peak is observed at *ħω* = 1.180–1.185 keV and asymmetric peak at 1.190 keV with shoulders on the low and high energy sides, the former carrying more spectral weight. Inspection of Fig. 2b,c, and the constant final state scans in ref. 23 reveal that the intensity of the decay from the 2p^5^5d^1^ intermediate state exceeds the emission intensity from the 2p^5^4f^ 8^ state at all incident x-ray energies, as expected since the latter intermediate state is dipole forbidden.

For the on-Laue case, the crystal is driven to the Laue [008] diffraction condition, and anomalous transmission is observed. Our sample suddenly becomes semi-transparent to x-rays. A *transmission* x-ray absorption spectrum can now be measured from the diffracted beam and we show the result in Fig. 2d. The quadrupole-excited 4f ^8^5d^0^ state is enhanced with respect to the off-Laue case. The modification in the spectrum is mainly due to difference in the relative weights of E1 and E2 absorption under the Borrmann condition^24^. The obtained spectrum is overall comparable to the room temperature results of Pettifer *et al.*^17^. However, the quadrupole enhancement effect on the x-ray absorption spectrum at ambient temperature is relatively modest.

Much more dramatically, the exit-surface on-Laue resonant x-ray emission spectra [Fig. 2e,f] exhibits a strikingly different behaviour from the off-Laue case. First of all, when the anomalously transmitted x-rays can illuminate the exit surface of the crystal, it becomes a strong emitter of x-rays, including L*α*_1_ radiation. As an even more spectacular phenomenon, now the resonantly excited L*α*_1_ line shape has changed dramatically. The dipole-excited intermediate state is diminished in spectral weight. In contrast, the quadrupole-excited intermediate state 4f^ 8^5d^0^ increases in relative intensity by a factor of ten due to the Borrmann effect. Figure 2f shows the on-Laue resonant x-ray emission spectrum to be compared to the off-Laue spectra of Fig. 2c. The maximum intensity of emission from the 2p^5^4f ^8^ intermediate state exceeds the one originating from the 2p^5^5d^1^ state. This is an important observation as the effect on x-ray absorption at room temperature is modest in comparison with cryogenic temperatures^17^,^24^.

The suppression of the emission related to the dipole-allowed intermediate state from the exit surface is not complete. We attribute the remaining dipole related emission to lattice vibrations and static sample disorder (e.g., surface roughness). X-ray emission from the entrance surface does not contribute to the exit-surface signal as it would have to travel at least 15 attenuation lengths before reaching the exit surface and the spectrometer. The on-Laue resonant x-ray emission map from the entrance surface was nearly identical with the one obtained in the off-Laue case and is not presented. The unchanged behaviour is to be expected on the entrance surface due to the dominance of the dipolar absorption of the *β*-branch while the *α*-branch experiences reduced absorption.

The observation of a novel type of x-ray emission excited by an anomalously transmitted x-ray beam opens up exciting possibilities to expand the capabilities of standing wave x-ray spectroscopy. Such experiments can be readily performed at synchrotron light sources using standard 1-eV resolution crystal spectrometers. This will lead to a more thorough understanding of x-ray absorption spectra and the electronic structure information derivable from the spectrum^24^. The observed effect could also be utilized in precision experiments measuring the energies and lineshapes of weak emission features lying close in emission energy to a strong dipole allowed RXES channel. Experiments with sub-1 eV resolution on low energy excitations, e.g. dd or crystal field excitations could exploit the anisotropy properties discussed by Tolkiehn *et al.*^24^. For example, their experimental and theoretical work on SrTiO_3_ demonstrates that utilising the (110) reflection in the perovskite structure, the quadrupolar transition rate to e_*g*_ states is enhanced while to the t_2*g*_ states it is suppressed. Furthermore, as pointed out by Pettifer *et al.*^17^, the enhancement of quadrupolar transitions could provide a method for quantitative characterization of the relative weights of the multipoles in the x-ray absorption spectra in various systems. This would be highly useful information in the interpretation of x-ray dichroic spectroscopies and characterising the nature of chemical bonds in e.g. oxides and related materials. For example, one can envision separating the effects of pd-hybridization and quadrupolar transitions at transition metal K edges. Since the Borrmann effect can be used to set up a standing wave of a desired periodicity and orientation, we suggest that site-selective spectroscopic information could be extracted via an analysis of standing wave measurements using appropriately chosen reflections.

## Discussion

We have demonstrated a significant relative intensity increase of resonant x-ray emission spectral features originating from quadrupole transition excited intermediate states in GGG. In contrast with the Borrmann spectroscopy introduced by Pettifer *et al.*, measuring the resonant x-ray emission spectrum under the Borrmann condition provides a stark contrast between the electric dipolar and quadrupolar transitions even at room temperature. Our results pave the way for many other interesting studies where weak, yet-to-be-exploited features in the x-ray absorption spectrum play a role in elucidating the electronic structure of complex materials, e.g., when the 3d or 4f electrons contribute to chemical bonding and magnetism. Future applications of dynamical diffraction phenomena in spectroscopy will greatly benefit from the improved brilliance of upcoming next generation light sources, as improvements in beam collimation whilst maintaining a sufficient monochromatic photon flux on the sample will facilitate manipulation of the x-ray wavefield in the sample. Our sample was a standard off-the-shelf commercial substrate and various crystals of 3d and 4f compounds of comparable quality are readily obtainable for further experiments. Our results show for the first time that x-ray standing wave methods^9^ can be extended to resonant x-ray emission spectroscopy, which is itself a widely used tool in materials, chemistry, and physics research^2^,^7^, and thus opens up new avenues for many research fields.

## Methods

We performed the experiment at the ID20 beamline of the European Synchrotron Radiation Facility. We used Si(111) crystals to monochromatize the incident x-rays, and a crystal spectrometer to record the emission spectra. The spectrometer employed two spherically bent Si (333) crystals in the Johann geometry with a bending radius of 2 m, and avalanche photodiodes as detectors. The energy resolution was ~1 eV. The photon flux on the sample was estimated to be 10^14^ s^−1^ (within 1 eV bandwidth at 7.25 keV). The incident beam divergence was <300 *μ* rad. The sample was a 150 *μ*m thick commercial Gd_3_Ga_5_O_12_ crystal with (001) surfaces and was kept in ambient temperature. We chose GGG for this demonstration due to availability of high quality single crystals and previously characterized x-ray spectra^17^,^23^. The sample was brought to the [008] Laue diffraction condition at the Gd L_3_ absorption threshold in the *σ* polarisation geometry as visualized in Fig. 1. The [008] reflection was chosen because it places the nodes of the *α*-branch on atomic planes occupied by the heavy Gd and Ga atoms^17^. We verified the Borrmann effect by observing the transmitted and Laue-diffracted beams using Si *pin* diodes behind the sample. We used similar diodes for total fluorescence yield x-ray absorption measurements as well. The RXES maps were measured by recording the resonantly excited Gd L*α*_1_ x-ray spectra with 61 distinct incident photon energies. The counting time for each point for the entrance-surface RXES spectra was 1 s, and 6 s for the exit-surface spectra. The exposure time accumulated for counting the RXES maps in Fig. 2b,e was 3 h and 9 h, respectively (not including dead time owing to motor movements). The pre-edge on-Laue RXES spectrum presented in Fig. 2f was obtained with a counting time of 30 s per measurement point.

## Additional Information

**How to cite this article**: Ruotsalainen, K. O. *et al.* Resonant X-ray emission with a standing wave excitation. *Sci. Rep.* **6**, 22648; doi: 10.1038/srep22648 (2016).
